# Women's autonomy in household decision-making: a demographic study in Nepal

**DOI:** 10.1186/1742-4755-7-15

**Published:** 2010-07-15

**Authors:** Dev R Acharya, Jacqueline S Bell, Padam Simkhada, Edwin R van Teijlingen, Pramod R Regmi

**Affiliations:** 1 Aberystwyth University, School of Education & Lifelong Learning, Old College, King Street, Aberystwyth SY23 2AX, UK; 2 University of Aberdeen, Immpact, Institute of Applied Health Sciences, Foresterhill, Aberdeen AB25 2ZD, UK; 3 University of Sheffield, School of Health and Related Research (ScHARR), Regent Court, 30 Regent Street, Sheffield S1 4DA, UK; 4 Bournemouth University, School of Health and Social Care, Maternal & Perinatal Health Research, Royal London House, Christchurch Road, Bournemouth BH1 3LT, UK; 5 University of Aberdeen, Department of Public Health, Foresterhill, Aberdeen AB25 2ZD, UK

## Abstract

**Background:**

How socio-demographic factors influence women's autonomy in decision making on health care including purchasing goods and visiting family and relatives are very poorly studied in Nepal. This study aims to explore the links between women's household position and their autonomy in decision making.

**Methods:**

We used Nepal Demographic Health Survey (NDHS) 2006, which provided data on ever married women aged 15-49 years (n = 8257). The data consists of women's four types of household decision making; own health care, making major household purchases, making purchase for daily household needs and visits to her family or relatives. A number of socio-demographic variables were used in multivariable logistic regression to examine the relationship of these variables to all four types of decision making.

**Results:**

Women's autonomy in decision making is positively associated with their age, employment and number of living children. Women from rural area and Terai region have less autonomy in decision making in all four types of outcome measure. There is a mixed variation in women's autonomy in the development region across all outcome measures. Western women are more likely to make decision in own health care (1.2-1.6), while they are less likely to purchase daily household needs (0.6-0.9). Women's increased education is positively associated with autonomy in own health care decision making (p < 0.01), however their more schooling (SLC and above) shows non-significance with other outcome measures. Interestingly, rich women are less likely to have autonomy to make decision in own healthcare.

**Conclusions:**

Women from rural area and Terai region needs specific empowerment programme to enable them to be more autonomous in the household decision making. Women's autonomy by education, wealth quintile and development region needs a further social science investigation to observe the variations within each stratum. A more comprehensive strategy can enable women to access community resources, to challenge traditional norms and to access economic resources. This will lead the women to be more autonomous in decision making in the due course.

## Background

Autonomy is the ability to obtain information and make decisions about one's own concerns [1]. It facilitates access to material resources such as food, land, income and other forms of wealth, and social resources such as knowledge, power, prestige within the family and community [2]. Women's autonomy in health-care decision-making is extremely important for better maternal and child health outcomes [3], and as an indicator of women's empowerment. Gender-based power inequalities can restrict open communication between partners about reproductive health decisions as well as women's access to reproductive health services. This in turn can contribute to poor health outcomes [4]. Evidence from other developing countries show that women's age and family structure are the strongest determinants of women's authority in decision making [5]. Older women and women in nuclear households are more likely than other women to participate in family decisions.

The socio-cultural context conditions the relationship of women's individual-level characteristics to decision-making, and autonomy is a key intervening mediator between women's status and reproductive outcomes [6]. Women have little autonomy in many cultures, so it is important to get (1) a better understanding of the determinants of their decision-making autonomy; (2) and variations across regions and socio-cultural contexts in the same country. Previous work has shown that women who have a significant say in reproductive matters tend to be more educated, spend more time on household economic activities and marry later [7]. Several other studies have also shown that the poor tend to be sicker and they utilise care facilities less frequently than their better-off counterparts [8-10]. An African study highlights that ethnicity plays a very important role in shaping a wife's decision-making authority and is even more important than other individual-level characteristics as a determinant of authority [11]. Another study emphasises that compared to their husbands' report, wives tend to under-report their household decision-making power. However, educated and employed partners are more likely to participate in the final decisions [12]. The level of women's autonomy also depends on whether wives or husbands are the respondents since it appears that the response categories do not have the same cognitive or semantic meanings for men and women [13]. Limitations to women's physical, sexual, economic, social and political autonomy also affect women's decision-making processes. Population and development programmes are most effective when steps have simultaneously been taken to improve the status of women in the decision making process [1].

In Nepal, as in most parts of South Asia, women commonly have less power and autonomy than men in making decisions about their own health care. Moreover, women often have unequal access to food, education, and health care, limited opportunities to earn incomes, restricted access to, and control over, productive resources, and very few effective legal rights [14]. Women's autonomy in decision making is associated with her ethnicity, deprivation level, urban/rural classification, education, and number of living children [15]. Nepalese women are further disadvantaged by a lack of awareness of opportunities and their legal rights. Their low social status has been identified as a barrier towards national health and population policy progress in Nepal [16,17]. Gender equity gives women both increased decision-making authority and more modern reproductive outcomes such as to reduce the desire for more children, increase contraceptive use and lower the level of 'unmet need' for contraception [18]. A Nepal Demographic Health Survey (NDHS) shows that women are generally less educated than men [19]. The survey reveals that 37% of currently married women participated in all four of the important household decisions that were investigated: their own health care, major household purchases, purchases of daily household needs and visits to her family or relatives; while 31% did not participate in any of these decisions.

## Methods

DHS surveys are nationally representative, population-based household surveys which provide accurate and internationally comparable data on health indicators in developing countries. DHS surveys are part of the worldwide DHS project whose objective is to improve population and health surveillance [20]. These are conducted around every five years in many low and middle-income countries; in all households from a large representative sample women aged 15-49 (and sometimes men aged 15-59) are interviewed. In Nepal, the DHS (2006) survey was conducted under the aegis of the Population Division of the Ministry of Health and Population and implemented by New ERA, a research organisation. Technical support for the survey was provided by Macro International Inc., and it was funded by the United States Agency for International Development (USAID). The survey provides information on fertility levels and determinants, family planning, fertility preferences, infant, child, adult and maternal mortality, maternal and child health, nutrition, knowledge of HIV/AIDS and women's empowerment including socio-economic and background characteristics of households [19]. The aim of this study is to establish the most important socio-background characteristics associated with women's decision-making power.

This study is secondary analysis based on the 2006 Nepal DHS data. The DHS conducted a nationally representative survey of 10,793 women aged 15-49 and 4,397 men aged 15-59; in total 8,257 married women were interviewed about their roles in decision-making. In Nepal, community norms and values affect individual behaviour, so women's age, employment (in the past 12 months), number of living children, residence type (urban or rural), ecological zone (Terai, hill or mountain) and development region were considered as socio-demographic variables. Wealth is described in DHS data by an asset score that is constructed using a principal component analysis of more than 40 asset variables collected by a household questionnaire-these include consumer goods, housing facilities and materials [21]. These asset scores are used to classify women into quintile groups according to the relative wealth of their household. Similarly, women's education has been consistently related to use of maternal and child health services, to positive health outcomes and to insist on participating in family decisions [22,23]. Information on level of schooling is collected for women and their partners, so wealth and education could both be included in the analyses. There is a strong sense of family togetherness in Nepal and individual identity is closely tied to that of the family; therefore making decisions often involves complex negotiations [24]. Hence, it is crucial to measure whether a woman is involved in the final decision-making process, using all these socio-background variables.

The original DHS questionnaire asked about four areas of women's autonomy in decision making. These are own health care, making major household purchases, making purchase for daily household needs and visits to her family or friends. Each question had six responses: (1) respondent alone; (2) respondent and husband/partner; (3) respondent and other person; (4) husband/partner alone; (5) someone else and (6) others. To create a binary variable for the analysis, we grouped the first three responses 1-3 (in which she has some power) and responses 4-6 (in which she has no say in the decision). The socio-background characteristics retrieved from the DHS data set are age, residence, ecological zone, development region, education and wealth quintile which are unchanged for our analysis. However, the background characteristics employment on past 12 months is re-categorised into three categories; not employed, employed for cash and employed not for cash. Similarly, number of living children is re-categorised into four categories 0, 1-2, 3-4 and 5+. Our multivariate regression explores whether socio-background characteristics are independently associated with women's autonomy in decision making. DHS granted permission to extract relevant data from the DHS web pages.

## Statistical Analysis

Analysis is conducted using SPSS version 17.0. Sample weights are used in order to adjust for the sample design; this ensures that the results are representative at a national level. The associations between the predictive (socio-background) factors and four outcome measures of women's decision-making are explored using cross-tabulations and the chi-squared test. Factors found to be significantly associated (at a 5% level; p < 0.05) with the outcome measures were then used in (a) bivariable and (b) multivariable logistic regression to generate odds ratios (ORs) and confidence intervals (95% CIs). To check the collinearity among predictive factors, the Pearson correlation coefficient *(r) *is calculated with p-value for significance. A backward-stepwise (BSTEP) method is used in multivariable logistic regression to determine the relative independent factor as a predictor of women's autonomy in decision-making. BSTEP regression starts with all the predictive factors included in the full starting model. It then removes the least significant covariate, that is, the one with the highest p-value, at each step, until all factors have been added. By scrutinising the overall fit of the model, variables will be automatically removed until the optimum model is found.

## Results

### Socio-background characteristics

Table 1 shows the percentage of women who report that they make specific household decisions alone or jointly with their husband. Cross-tabulation result shows that socio- background characteristics are significantly associated with all four types of women's decision making. Of those total respondents, almost half (47.1%) of ever-married women took decisions on their own health care alone or jointly with their husband. This proportion compares with 52.8% on making major household purchases, 57.6% for making daily household purchases and 56.6% for visits to family/friends. Participation in own health care decision making gradually increased by age, from 17% among women aged 15-19 to 60.3% in middle-aged women (45-49). Similar age-related decision-making power can be observed for major household purchases (15.5%-71.3%), daily purchases (18.0%-74.6%) and visits to family and friends (20.1%-77.0%). Women in paid employment also have a higher say in decision making.

**Table 1:** Percent of women's participation in decision making

Background characteristics	Own health care (%)	Major household purchases (%)	Purchases for daily household needs (%)	Visits to her family or relatives (%)	Number (n^w^)
**Age**					
15-19	17.0	15.5	18.0	20.1	784
20-24	37.1	35.2	38.8	40.2	1,606
25-29	48.5	51.2	58.0	54.8	1,664
30-34	52.0	62.3	67.9	64.2	1,265
35-39	55.2	65.3	72.0	68.2	1,135
40-44	58.6	71.7	75.3	75.0	1,016
45-49	60.3	71.3	74.6	77.0	788
**Employment (past 12 months)**					
Not employed	43.5	49.7	54.5	51.9	1,376
Employed for cash	59.6	72.7	77.4	75.4	2,438
Employed not for cash	41.4	42.8	47.8	47.6	4,443
**Number of living children**					
0	21.9	21.3	23.0	26.1	860
1-2	45.2	48.3	52.8	52.3	3,364
3-4	53.7	63.5	68.5	66.2	2,831
5+	55.0	62.5	70.3	67.7	1,202
**Residence**					
Urban	54.6	63.9	71.8	67.7	1,226
Rural	45.8	50.8	55.2	54.6	7,031
**Ecological zone**					
Mountain	43.5	47.1	50.1	50.7	586
Hill	50.5	57.3	63.6	63.4	3,402
Terai	44.9	49.9	53.9	51.9	4,269
**Development region**					
Eastern	46.3	53.8	61.0	57.6	1,757
Central	46.7	54.5	61.5	56.9	2,736
Western	52.5	51.0	56.3	56.9	1,602
Mid-western	45.8	50.0	56.5	60.6	976
Far-western	43.1	51.8	46.4	50.5	1,187
**Education**					
No Education	47.4	54.9	59.3	58.3	5,110
Primary	44.7	49.2	54.3	52.0	1,404
Some secondary	45.0	44.0	50.8	51.2	1,197
SLC and above/higher	55.8	60.7	65.4	63.4	547
**Wealth quintile**					
Lowest	45.7	51.9	56.0	57.1	1,537
Second	49.6	52.7	57.0	56.3	1,642
Middle	42.7	45.5	49.3	49.6	1,747
Fourth	44.6	49.7	55.3	53.7	1,640
Highest	53.0	64.0	70.6	66.3	1,692
**Total**	**47.1%**	**52.8%**	**57.6%**	**56.6%**	**8,257**

Notes: All chi-square (χ2) test showed statistically significant association with p < 0.05 at 95% CI; n^w ^= weighted totals

Women with more living children (5+) have greater participation in decision making for each outcome variable. Making major household purchases is the only exception, as women with three or four children had a slightly higher participation rate (63.5%) than those with five or more children (62.5%). Women from urban areas and the hill region, those in highest wealth quintile and those with levels higher than SLC (School Leaving Certificate) also have a greater say in the decision-making process. Interestingly, women with no education have a higher say compared to those primary or some secondary education for all four outcome variables. Development regions and women's response shows mixed variations across the outcome variables.

### Collinearity and bivariate analysis

The value from Pearson correlation coefficient *(r) *shows that while many of the covariates are correlated to some degree only age and parity are correlated with a coefficient >0.5 (actual value 0.65). Each of the four outcome measures of women's autonomy in decision making varies significantly according to socio-background characteristics (Table 2). Women's age shows a positive association with these outcome variables. An exception is the age range 45-49 in major household purchases; being older is more likely to provide autonomy in decision making than being younger.

**Table 2:** Bivariate analysis of women's participation in decision making and socio-background characteristics

Socio-demographic Characteristics	Own health care		Major household purchases		Purchases daily household needs		Visits to her family or relatives	
	Odds Ratios	95% CI	Odds Ratios	95% CI	Odds Ratios	95% CI	Odds Ratios	95% CI
**Age**								
15-19	1.0		1.0		1.0		1.0	
20-24	2.88***	(2.33, 3.56)	2.95***	(2.37, 3.67)	2.88***	(2.34, 3.55)	2.67***	(2.18, 3.26)
25-29	4.60***	(3.73, 5.67)	5.70***	(4.60, 7.08)	6.29***	(5.12, 7.74)	4.82***	(3.95, 5.89)
30-34	5.28***	(4.25, 6.56)	8.97***	(7.17, 11.23)	9.65***	(7.77, 11.99)	7.13***	(5.78, 8.79)
35-39	6.02***	(4.83, 7.50)	10.22***	(8.13, 12.84)	11.72***	(9.37, 14.65)	8.55***	(6.90, 10.60)
40-44	6.91***	(5.52, 8.65)	13.77***	(10.87, 17.45)	13.88***	(11.01, 17.49)	11.95***	(9.54, 14.97)
45-49	7.43***	(5.88, 9.40)	13.48***	(10.53, 17.27)	13.39***	(10.50, 17.07)	13.35***	(10.49, 16.99)
**Employment (past 12 months)**								
Not employed	1.0		1.0		1.0		1.0	
Employed for cash	1.91***	(1.67, 2.19)	2.69***	(2.34, 3.08)	2.84***	(2.46, 3.28)	2.84***	(2.46, 3.26)
Employed not for cash	0.91	(0.81, 1.03)	0.75***	(0.67, 0.85)	0.76***	(0.67, 0.86)	0.84**	(0.74, 0.95)
**Number of living children**								
0	1.0		1.0		1.0		1.0	
1-2	2.93***	(2.46, 3.49)	3.45***	(2.89, 4.12)	3.75***	(3.16, 4.46)	3.09***	(2.62, 3.65)
3-4	4.13***	(3.46, 4.93)	6.43***	(5.37, 7.70)	7.29***	(6.10, 8.71)	5.53***	(4.66, 6.57)
5+	4.35***	(3.57, 5.29)	6.17***	(5.05, 7.55)	7.92***	(6.48, 9.69)	5.92***	(4.87, 7.19)
**Residence**								
Urban	1.0		1.0		1.0		1.0	
Rural	0.70***	(0.62, 0.79)	0.58***	(0.51, 0.66)	0.48***	(0.42, 0.55)	0.57***	(0.50, 0.65)
**Ecological zone**								
Mountain	1.0		1.0		1.0		1.0	
Hill	1.32**	(1.10, 1.57)	1.50***	(1.26, 1.79)	1.74***	(1.46, 2.07)	1.68***	(1.41, 2.01)
Terai	1.05	(0.89, 1.26)	1.12	(0.94, 1.33)	1.16	(0.98, 1.38)	1.04	(0.88, 1.24)
**Development region**								
Eastern	1.0		1.0		1.0		1.0	
Central	1.01	(0.90, 1.14)	1.02	(0.91, 1.15)	1.01	(0.90, 1.15)	0.97	(0.85, 1.09)
Western	1.28***	(1.12, 1.46)	0.89	(0.77, 1.02)	0.82**	(0.71, 0.94)	0.97	(0.84, 1.11)
Mid-western	0.98	(0.83, 1.14)	0.85	(0.73, 1.00)	0.82*	(0.70, 0.97)	1.13	(0.96, 1.32)
Far-western	0.88	(0.75, 1.02)	0.92	(0.79, 1.06)	0.55***	(0.47, 0.64)	0.75***	(0.64, 0.87)
**Education**								
No Education	1.0		1.0		1.0		1.0	
Primary	0.89	(0.79, 1.01)	0.79***	(0.70, 0.89)	0.81**	(0.72, 0.91)	0.77***	(0.68, 0.87)
Some secondary	0.98	(0.87, 1.10)	0.73***	(0.65, 0.82)	0.79***	(0.71, 0.89)	0.82**	(0.73, 0.92)
Higher (SLC and above)	1.58**	(1.20, 2.07)	1.41*	(1.07, 1.86)	1.34*	(1.01, 1.78)	1.38*	(1.04, 1.84)
**Wealth quintile**								
Poorest	1.0		1.0		1.0		1.0	
Poorer	1.16*	(1.01, 1.34)	1.03	(0.89, 1.18)	1.04	(0.90, 1.19)	0.96	(0.84, 1.11)
Middle	0.88	(0.77, 1.01)	0.77***	(0.67, 0.89)	0.76***	(0.66, 0.87)	0.73***	(0.64, 0.84)
Richer	0.95	(0.83, 1.10)	0.91	(0.79, 1.05)	0.97	(0.84, 1.12)	0.87	(0.75, 1.00)
Richest	1.33***	(1.16, 1.53)	1.65***	(1.43, 1.90)	1.88***	(1.63, 2.18)	1.47***	(1.28, 1.70)

Notes: OR = odds ratio; 95% CI = 95% confidence interval; *p < 0.05; **p < 0.01; ***p < 0.001.

Women's employment shows a significant relationship with all four outcome measures. Women who work for cash are more likely to participate in health care decision making, making major household purchases, daily household purchases and visits to her family or friends than those who are not employed and those who do not work for cash. Women's increased number of living children has a strong positive association with all the outcome measures in decision making. Women's residence has also a strong association with all four outcome measures in decision making. Rural women are less likely to be autonomous (p < 0.001) in decision making compared to their urban counterparts.

Women from the hill region are more likely to have autonomy in decision making in all four outcome measures. Compared to the mountain region, women from the Terai (in south of Nepal) are more likely to be autonomous in decision making; however it is not significantly associated (p > 0.05) to all four outcome measures. The development region shows fewer significant relationships in women's decision making. Women from the western development region are more likely to participate in health-care decision making compared to all other regions. In contrast, women from the western, mid-western and far western region are less likely to participate in decision making on daily household needs. Furthermore, a significantly lower proportion of women from the far-western region reported involvement in decision making around visiting family or relatives (p < 0.001).

Women who are educated to SLC level and above are more likely to participate in all four outcome measures. Interestingly, women with primary and some secondary level education are less likely to participate in decision-making around major household purchases, daily household purchases and visits to her family or friends compared to women without education. The richest women are more likely to participate in decision making in all four outcome measures (p < 0.001). Conversely, middle-class women are significantly less likely to participate in making major household purchases, daily household purchases and visits to her family or relatives compared to all wealth quintiles.

### Multivariate analysis

In this analysis age, employment and number of living children are highly significant to women's autonomy in decision making (Table 3). Age shows a positive relationship to decision making in all four outcomes; younger women are less likely to participate in decision making than older women. Women working for cash are more likely to participate in decision making in all four outcomes (p < 0.001) compared to the women who are not employed or paid in kind. The number of living children a woman has also shows a strong positive relationship with decision-making participation. The more children women have, the more likely they participate in decision making in all four outcomes. From the residential viewpoint, rural women are less likely to participate in the decision-making process.

**Table 3:** Final backward stepwise multivariate analysis model assessing determinants of Nepalese women's autonomy in decision making

Socio-demographic characteristics		Outcome-1 (own health care)		Outcome -2 (major household purchases)		Outcome -3 (daily household purchases)		Outcome -4 (visits to family and friends)	
		OR	95%CI	OR	95%CI	OR	95%CI	OR	95%CI
**Age**	15-19	1.0		1.0		1.0		1.0	
	20-24	2.19***	(1.74, 2.76)	2.06***	(1.62, 2.61)	1.92***	(1.52, 2.42)	1.95***	(1.56, 2.44)
	25-29	3.23***	(2.54, 4.10)	3.37***	(2.63, 4.32)	3.57***	(2.81, 4.55)	3.09***	(2.44, 3.90)
	30-34	3.66***	(2.84, 4.72)	5.32***	(4.09, 6.93)	5.18***	(4.00, 6.72)	4.47***	(3.47, 5.75)
	35-39	4.48***	(3.44, 5.83)	6.80***	(5.16, 8.96)	6.93***	(5.27, 9.11)	6.03***	(4.62, 7.87)
	40-44	5.22***	(3.98, 6.83)	9.42***	(7.08, 12.53)	8.11***	(6.12, 10.76)	8.67***	(6.57, 11.43)
	45-49	5.82***	(4.38, 7.73)	9.42***	(6.97, 12.71)	7.72***	(5.73, 10.40)	9.98***	(7.42, 13.43)
**Employment**	Not employed	1.0		1.0		1.0		1.0	
	Employed for cash	1.41***	(1.22, 1.63)	1.72***	(1.47, 2.01)	1.97***	(1.67, 2.32)	1.86***	(1.59, 2.18)
	Employed not for cash	0.73***	(0.63, 0.85)	0.53***	(0.45, 0.62)	0.61***	(0.52, 0.72)	0.57***	(0.48, 0.66)
**Number of living children**	0	1.0		1.0		1.0		1.0	
	1-2	1.81***	(1.48, 2.21)	1.93***	(1.56, 2.37)	2.12***	(1.73, 2.61)	1.78***	(1.46, 2.17)
	3-4	1.97***	(1.58, 2.45)	2.26***	(1.79, 2.84)	2.72***	(2.16, 3.42)	2.06***	(1.65, 2.57)
	5+	1.81***	(1.42, 2.33)	1.65***	(1.27, 2.14)	2.41***	(1.86, 3.14)	1.62***	(1.25, 2.10)
**Residence**	Rural	0.80**	(0.69, 0.94)	0.87	(0.73, 1.02)	0.69***	(0.58, 0.83)	0.77**	(0.65, 0.91)
**Ecological zone**	Mountain	1.0		1.0		1.0		1.0	
	Hill	1.10	(0.90, 1.33)	1.37**	(1.12, 1.68)	1.48***	(1.21, 1.82)	1.54***	(1.26, 1.88)
	Terai	0.91	(0.75, 1.10)	0.89	(0.73, 1.09)	0.91	(0.74, 1.11)	0.86	(0.70, 1.05)
**Development region**	Eastern	1.0		1.0		1.0		1.0	
	Central	0.95	(0.83, 1.08)	0.85*	(0.74, 0.98)	0.82**	(0.71, 0.94)	0.79**	(0.69, 0.91)
	Western	1.39***	(1.20, 1.61)	0.92	(0.78, 1.07)	0.78**	(0.66, 0.92)	0.97	(0.83, 1.14)
	Mid-western	1.07	(0.90, 1.26)	0.95	(0.79, 1.13)	0.86	(0.72, 1.03)	1.26*	(1.05, 1.52)
	Far-western	0.96	(0.82, 1.13)	1.11	(0.93, 1.31)	0.56***	(0.47, 0.67)	0.85	(0.72, 1.01)
**Education**	No Education	1.0		1.0		1.0		1.0	
	Primary	1.25**	(1.09, 1.43)	1.21**	(1.05, 1.40)	1.24**	(1.07, 1.44)	1.15*	(1.00, 1.33)
	Some secondary	1.52***	(1.32, 1.76)	1.14	(0.98, 1.33)	1.26**	(1.08, 1.47)	1.35***	(1.16, 1.57)
	Higher (SLC and above)	1.85***	(1.36, 2.52)	1.28	(0.92, 1.78)	1.11	(0.79, 1.57)	1.32	(0.94, 1.84)
**Wealth quintile**	Poorest	1.0		1.0		1.0		1.0	
	Poorer	1.14	(0.98, 1.32)	1.02	(0.87, 1.20)	1.02	(0.86, 1.20)	0.99	(0.84, 1.16)
	Middle	0.83*	(0.71, 0.97)	0.72***	(0.61, 0.85)	0.68***	(0.58, 0.81)	0.72***	(0.61, 0.85)
	Richer	0.77**	(0.65, 0.91)	0.76**	(0.64, 0.90)	0.75**	(0.63, 0.89)	0.72***	(0.61, 0.86)
	Richest	0.75**	(0.62, 0.91)	1.02	(0.83, 1.24)	1.05	(0.85, 1.29)	0.85	(0.69, 1.04)

Notes: OR= odds ratio; 95% CI = 95% confidence interval; *p < 0.05; **p < 0.01; ***p < 0.001

In outcome-1, women from the hill region have a higher participation in decision making around their own health care than those from the mountain and Terai regions; however it is not statistically significant. Women from the western development region have significantly greater influence in health-care decision making for themselves. Education also affects women's ability to make their own decisions. Women with more schooling (SLC and above) are more likely to make decision about their own health care compared to those who have some secondary or primary or no education. It is interesting to note that the richest women are significantly less likely to participate in decision making (p < 0.01) about their own health care compared to all other income groups after adjustment for other factors.

Women's participation in decision making to make major household purchases also has a strong significant association with socio-background characteristics in outcome-2. Here age, employment, number of living children, ecological zone (hill), development region (central), education (primary level) and wealth quintile (middle and richer) are significantly associated with the outcome measure, but rurality is not associated. In outcome-3 age, employment, number of children, ecological zone (hill) and education (some secondary) have strong odds ratios (ORs) and are significantly associated with the outcome measure. Women from the far western region are the least likely to take part in decision making compared to other regions. The association between schooling level and deciding about daily household purchases yields a non-significant result (p > 0.05) with higher education (SLC and above), however it is significant with primary and having some secondary education. It is clear that women's schooling plays a significant role in taking part in the decision- making; however our finding has created a complex scenario which needs further social-science investigation. Women with middle-wealth quintile are also less likely to take part in decision making compared to both richer and the poorest women.

Outcome-4 shows that an increase in age is directly associated to an increase in odds ratios (ORs), which examine the likelihood of women's participation in making decisions to visit her family and friends. As women gets older, they are more likely to take part in the decision making process to visit her family and friends (p < 0.001). Women employed for cash and having 3-4 living children also have a greater say in the decision-making process. Residence (rurality), development region (central) and wealth quintile (middle and richer) have a negative association with the outcome measures; these women are less likely to participate in decision making to visit family and friends.

## Discussion

Increased age, paid employment and having a greater number of living children are all positively associated with women's autonomy in decision making in all four outcomes. Residence (rurality) is less likely to do so in neither the bivariate or multivariate analysis in all outcome measures. In both analyses, women from the hill region are more likely to be autonomous in decision making, except in outcome-1 in the multivariate analysis (p > 0.05). In bivariate analysis, the development region shows a non-significant result for making major household purchases; however women from the central region are less likely to do so and to decide about purchase daily household needs in the multivariate analysis. Women from the far western region are less likely to be involved in the decision to visit family or relatives in the bivariate analysis, and this pattern has shifted somewhat in the multivariate analysis. Women with more schooling (SLC and above) are more likely to be autonomous in own health care in the both analyses; but they are joined by women with primary and some secondary education in the multivariate analysis. Women with primary education are less likely to decide about major household purchases in the bivariate analysis, while they are more likely to do so in the multivariate analysis.

Women with some secondary (less likely) and more schooling (more likely) are also significantly associated with major household purchase in bivariate analysis, while multivariate analysis does not show such significance. Women with primary and some secondary education are more likely to be autonomous in making daily household purchases and visiting family and friends in multivariate compared to the bivariate analysis. The richest women are significantly more likely to make decisions in all four types of outcome measures in bivariate analysis. However, the multivariate result shows that they are less likely to make decisions in the outcome-1. Poorer women are significantly more likely to be autonomous to make decisions about own health care in the bivariate, while it is non-significant in the multivariate analysis.

### Age and number of living children

There is a significant positive association between women's age and autonomy in decision making among all four measures. This association also exists for the number of living children; women with more living children are more likely to take part in decision making. Autonomy is not a homogenous construct that is represented accurately by a single measure. In Nepal, Bangladesh and India, as women get older they gain autonomy in household decision making [25]. A newly married daughter-in-law has less decision making power in the household and she is expected to perform household duties under the supervision of her mother-in-law who is the primary decision maker [26]. Some possible factor behind this autonomy is that the older women move out of extended family responsibility, or that women fear that attempts to discuss issues around decision-making to control their own sexuality and reproduction with their husband may lead to aggression [27]. The issue of security and fulfilment of desire also becomes less importance as women gets older and lose contact with their natal kin and become more likely to be independent in decision making. Nevertheless, in some Asian countries, such as Sri Lanka, there is a more collective responsibility around decision-making between men and women in 60.3% of the households [28].

### Employment

Women's ability to make household decisions is enhanced while they are working. Traditionally Nepalese women were not expected to be in paid employment, so those who work for money used to be from poor families or they work in the household for their family's survival. In addition, some women are employed but not for cash (e.g. *kamaiya, hali*), they work for landlords (*jamindaar*), who own large areas of farm land. These women work throughout the year while others work seasonally such as paddy cropping *(dhaan ropne)*, wheat harvesting *(gahun kaatne)*, or herding *(gothaalo jaane)*. They work for subsistence, e.g. food and clothes, and they are mostly from so-called lower casts, and have little decision-making power. Their economic condition stops them from making large or even daily household purchases. The relationship between employment and women's autonomy in decision making appears straightforward. It is clearly shown that women in paid employment are significantly more likely to report to participate in the final decision making compared to those women who are not in paid employment [12].

In Nepal, men often control the household's cash, making it difficult for women to pay for health care or for transportation to health-care facilities. This ultimately limits women's participation in decision making regarding their own health care, household purchases or visiting family or friends. Paid employment appears to empower married women to develop thinking towards participation in decision making. Women's work in the home is not a substitute for work outside the home for the women who desire employment [29]. Further analysis into the benefits and liabilities of women's employment and unemployment in women's participation in decision making is necessary.

### Residence

Rural women are significantly less likely to take part in decision making than urban women. The role of place in decision making is now widely recognised beyond the physical environment, which affects the health of people living there. Individual time-space circumstances interact with conditions in the local area, particularly in communities characterised by poverty and social exclusion [30]. In Nepal, about 80% of the population live in rural areas, generally within large families. Many are landless, have very small landholdings and are from specific ethnic minority groups such as low caste (*dalit*) and indigenous peoples (*janajati*). Geographic isolation of the rural population and their resulting exclusion from basic social services and economic opportunities is a root cause of poverty in Nepal. Many rural women live in severe poverty without any means of improving conditions for themselves and their families, which hinder them from making purchases for household needs. A South Asian study has also mentioned that rural women are less likely to be involved in decision making than urban women [25]. However, in recent years many community-based programmes have been initiated to raise incomes of the rural poor women, connect them to markets and provide economic opportunities through development of rural infrastructure [31]. Such programmes help women to gain access to new social networks and promote their social status, leadership roles, and autonomy in decision making.

### Ecological zone

Topographically, Nepal is divided into three ecological zones e.g. mountain (35%) in the northern region, hill (42%) in the mid region and the Terai (23%) plane in the south. The mountain region is the harsh terrain where transportation and communication facilities are very limited, and only about seven percent of the total population lives here. In contrast, the hill region is densely populated and contains about forty four percent of the total population. The country's most fertile and urbanised area, Kathmandu valley, lies in this region. Unlike the mountain and hill, the terai region in the south is relatively flat, where transportation and communication facilities are more developed. About forty four percent of various types of people live in the Terai, including ethnic groups and others that have roots in India [32]. Our finding shows that the women who live in hilly areas are more likely to participate in decision making compared to the mountain and Terai region women. This suggests that women who live in hilly areas have more autonomy towards the decision making process and their husbands are more likely to support them. Nepal's Terai region is adjacent to the north of India. Women's decision making, freedom from threatening relations with husband, mobility and access to and control over economic resources is highly constrained in north India [33]. A study has clearly noted that the practice of seclusion of women *(pardah) *is prevalent in Terai region especially for newly married women [24], while women in hills and mountains have more freedom of mobility and greater access to familial and economic resources after marriage.

### Development region

Administratively, Nepal is divided into five development regions- Eastern, Central, Western, Mid-western and Far-western [34]. However, little research has been conducted on development regions, women's health care and autonomy in decision making. The study findings are varied according to regions and it is hard to come up with possible explanations. For instance, western and mid-western region women have more freedom to make a decision in their own health care. Their role may be limited to making a decision on major household purchase and daily household purchases. However, this is not enough of a rigorous explanation to understand the root cause of such variations. There is very little known or understood about the influences of regions and women' decision making process in Nepal. An India study suggests that the southern region women have more exposure to the outside world, a greater voice in family life and more freedom of movement than do those of the north [22,35]. Nepal is largely gender stratified by inheritance and hierarchical relations, and the pattern of female autonomy varies within the regions considerably. Region plays the major conditioning role in women's autonomy in their lives [33]. The dominant behaviour and norms in the region's social system and women's exposure to the outside world provides them more freedom. So, further analysis is needed into whether development region leads to more autonomy for women or other confounding factors affect autonomy. Future research should look at women's autonomy changes across regions.

### Education

Highly educated women are more likely to take part in decision making in their own health care. Traditionally, older women (mothers-in-law) make decisions about young women's health care in Nepal [36]. However, perhaps young educated women subtly influence their mothers-in-law's decisions and introduce innovative ideas on decision making at the same time. Education may impart feelings of self-worth and self-confidence, which are more important features in bringing about changes in health-related behaviour than exposure to relevant information [37]. Nevertheless, greater education may reduce the power differential between providers and clients and lower women's unwillingness to seek care. Improvement in educational level and economic conditions is not sufficient to address the gender inequality in South Asia. The latest Human Development Report (2009) clearly describes that Nepal's GDI rank (gender-related development index) is 112^th ^out of 155 countries in the world [38]. There has been an increase in the enrolment of female pupils in Nepalese schools [31], but gender equity has to be incorporated as a core value at the policy level, if education aims to promote the autonomy of women [39]. It has to build up women's capacity to control resources and promote positive self-perceptions, self-confidence, awareness of rights and the ability to achieve them. Supporting community-based programmes increases poor women's participation to develop their capacity, to raise awareness, to build confidence and to develop leadership.

### Wealth quintile

The varied result in decision making suggests that there are other factors which explain the crude association between wealth and women's autonomy in decision making. Women's economic status in the household emerged as an important factor associated with their autonomy in decision making. It seems that an important aspect of this difference lies in the perceptions of household members, particularly in older women, regarding the need of autonomy for women. It also indicates that as the women gets richer; they are less likely to take part in decision making. The ownership and control of property is one of the most critical contributors to the gender gap in economic well-being, social status, and empowerment [40]. In Nepal, lack of women's power in the household decision-making process may have contributed to insufficient health care seeking behaviour. About 80% of Nepal's population still lives in rural areas, characterised by small landholdings, rapid population growth and a fragile ecology, resulting in chronic poverty in many parts of the country [31]. The gender empowerment measure (GEM) determines whether women take an active part in economic and political life. It exposes that Nepal ranks 83^rd ^out of 109 countries in the GEM, highlighting there are inequalities in opportunities among women in selected areas [38].

There are some limitations to this study. In general, men head and control the family unit in Nepalese societies. So, the possibility is that joint decisions have been reached which really meant convincing women to agree with the male head of the household. There is also the probability of recall and interviewer bias in the data set. This is a quantitative survey examining a wide variety of issues so it lacks in-depth information. Since we have conducted multiple logistic regression analysis, we have tried to address the problem of confounding. Intra-household attentions are explained to improve husband-wife communication which may strengthen women's influence within households for decision making [41]; however this study lacks such information. It is advised to construct an index combining the four binary variables and use that in the Ordinary Least Squares (OLS) regression. However, the method requires careful investigation and it is considered as a suggestion for future research.

## Conclusions

Many factors affect the ability of women to take part in the decision-making process in the household. Some of these factors relate to the type of decision that is taken and some to the background of the women. The third millennium development goal (MDG) aims to promote gender equality and empower women. It emphasises to increase financial resources to accelerate the goal that equally benefit and empower women and girls [42]. Many intervention programmes exist to improve women's household position in Nepal; however their situation still appears as bleak. Women from middle and richer class have the least decision-making power, which suggests involving them in education and decent employment to lessen their dependency on the family members and husband/partner. In the household, husband-wife relations are central to women's autonomy in decision making, and improved communication between them can deserve sustained support. Women are excluded from decision-making by more than just lack of education [43]. Employment and education have always empowered women and brought a positive impact on decision making [44], including reducing the inequalities among men and women. One effective method to do so is to incorporate the notion of empowerment in school curricula [45]. Attention should also be given to those women who do not attend school, through non-formal education. A curriculum for such programmes should be developed with a clear policy framework to reduce differences in education and employment between men and women.

Remote and rural women's involvement in income generation activities is another aspect of women's empowerment, and it can be done by supporting them in entrepreneurship, including improved access to property and economic assets, training, microfinance and markets. There is a need for a specially designed empowerment programme for women in the Terai, where gender-stratified setting is high and women's low autonomy is largely the result of traditional factors. Above all, it is strongly argued that women's autonomy should enhance not just her education and employment [6]. Somewhat, a more comprehensive strategy must be sought that could raise women's gender consciousness, enable them to access community resources and provide support for challenging traditional norms which cause gender inequalities [31,46,47]. Nepalese programme and policy initiatives should develop a clear policy foundation that should be crucial to empower women to take part in decision-making processes in the household. Moreover, enhancing their access to and control over economic resources and enabling them to establish and realise their rights are also essential means to empower them to be more autonomous in decision making.

## Competing interests

The authors declare that they have no competing interests.

## Authors' contributions

DRA and PS jointly developed the principal idea for the analysis. DRA and JSB obtained the data and did the statistical analysis. DRA further analysed, reviewed and drafted the paper. JSB, PS, EvT and PRR supervised the data and commented on the draft. All authors read and made substantial contributions to draughts and approved the final manuscript.
